# Human Papilloma Virus Vaccination and Oropharyngeal Cancer: Knowledge, Perception and Attitude among Italian Pediatric Dentists

**DOI:** 10.3390/ijerph19020790

**Published:** 2022-01-11

**Authors:** Tiziana Cantile, Stefania Leuci, Andrea Blasi, Noemi Coppola, Roberto Sorrentino, Gianmaria Fabrizio Ferrazzano, Michele Davide Mignogna

**Affiliations:** 1 Oral Medicine Unit, Department of Neurosciences, Reproductive and Oral Sciences, “Federico II” University of Naples, 80131 Naples, Italy; tizianacantile@yahoo.it (T.C.); andreablasi79@gmail.com (A.B.); noemi.coppola@unina.it (N.C.); mignogna@unina.it (M.D.M.); 2 Division of Prosthodontics and Digital Dentistry, Department of Neurosciences, Reproductive and Oral Sciences, “Federico II” University of Naples, 80131 Naples, Italy; errestino@libero.it; 3 Department of Neuroscience, Reproductive and Oral Sciences, School of Paediatric Dentistry, “Federico II” University of Naples, 80131 Naples, Italy; gianmariafabrizio@yahoo.it; 4 Unesco Chair in Health Education and Sustainable Development, Paediatric Dentistry Section, “Federico II” University of Naples, 80131 Naples, Italy

**Keywords:** HPV vaccination, HPV-related oropharyngeal cancer, pediatric dentistry, knowledge, perception, attitude

## Abstract

Background: Pediatric dentists could play a key role in the prevention of human papilloma virus (HPV)-related oropharyngeal cancer (OP-cancer). The aim of this study was to assess knowledge, perception, and attitude on HPV-related OP-cancer, HPV infection, and HPV vaccination among Italian pediatric dentists. Methods: A cross-sectional study was conducted. Pediatric dentists received, by email, a link to participate in the questionnaire online. The questionnaire comprised four parts: (i) demographic information, (ii) knowledge on HPV-related OP-cancer, HPV infection, and HPV vaccine, (iii–iiii) perceptions and attitude on HPV-related OP-cancer, HPV infection, and HPV vaccine. Data were statistically analyzed with Kruskal–Wallis and Mann–Whitney test and Pearson’s chi-square test. Results: A total of 271 pediatric dentists completed the questionnaire. Results showed a good overall knowledge; a positive perception of their role in HPV disease prevention; a good attitude in discussing sensitive topics; a need for acquiring more information about HPV’s connection to cancer, HPV infection, and HPV vaccine. Conclusions: Improving educational training programs, as well as informing about prevention of HPV-related OP-cancer, will place pediatric dentists in the front line of HPV diseases primary prevention.

## 1. Introduction

Oropharyngeal cancer (OP-cancer) is a serious global health problem, with an estimated 710,000 new cases and 350,000 deaths annually [1] and an overall 5-year survival rate equal to 65% [2]. In the last decades, OP-cancer was mainly diagnosed in adult subjects with a positive history in relation to the use of tobacco and alcohol. Nowadays, although per capita tobacco and alcohol consumption has declined, the incidence of OP-cancer has increased significantly [3].

Furthermore, white men with no or little tobacco and alcohol exposure aged between 40 and 50 years are currently described as typical patients affected by OP-cancers. This evidence can be explained considering that the disease is now increasingly attributed to human papilloma virus (HPV) infection [4]. 

HPV is the most common infective agent transmitted in sexually active individuals, and HPV infection can be early-acquired during first sexual intercourses. It is estimated that about 70% of sexually active men and women will harbor an HPV infection at some point in their lifetime [5].

Persistent infections with a high-risk HPV strain can lead to different malignancies (cervical, anal, penile, vaginal, vulvar, and OP-cancers) [6]. Certain sexual behaviors, including oral sex practices, high number of lifetime sex partners, age at sexual debut less than or equal to 18 years, and same-sex intercourse, represent an important risk factor for HPV-related OP-cancer [4].

The viral etiology of HPV-related cancers allows to develop an effective immunization strategy, thanks to the availability of three different types of vaccines (bivalent, quadrivalent, and nonavalent vaccines). In fact, HPV infection is a preventable disease by vaccination [7]. The ideal timing for HPV vaccination is prior to sexual debut, because clinical trials on HPV vaccine suggest that its efficacy is highest in HPV-naïve individuals [8]. In fact, routine vaccination is recommended for 11–12-year-old adolescents of both sexes, before the beginning of any sexual activity and possible exposure to HPV [9]. In addition, the immunogenic response to vaccines is enhanced in younger subjects [10].

In Italy, HPV vaccination has been recommended to young girls since 2008 and to young boys since 2017 [11]. Not all vaccines are available in all regions. In the absence of limitations related to availability and costs, the nine-valent vaccine is recommended [11].

The bivalent vaccine provides two doses in subjects aged between 9 and 14 years and three doses in subjects over 14 years of age. The quadrivalent vaccine provides two doses in individuals from 9 to 13 years and three doses in individuals aged 14 years and over. The nonavalent vaccine provides two doses in individuals from 9 to 14 years of age and three doses in individuals aged 15 years and over at the time of the first administration [11].

However, although HPV vaccination can be considered an effective anticancer preventive strategy, its administration remains at a suboptimal level [6,9]. In particular, despite the high efficacy and safety of the HPV vaccines, in Italy, the average vaccination coverage for HPV in girls is below the optimal threshold set by the National Vaccinal Prevention Plan (95% at the 12th year of life). In fact, in 2019, based on a national survey on adolescents, it was estimated that vaccine coverage ranged between 17.31% and 72.36% among females aged 12 (year of birth: 2007), depending on different geographic locations. Furthermore, the average vaccination coverage for HPV in boys is far below the optimal threshold set by the National Vaccinal Prevention Plan 2017–2019 (95% in 2019) [11,12].

As the vaccination campaign is targeted toward pre-adolescents, parental acceptance of the HPV vaccine is required for administration. Several parental barriers to HPV vaccine uptake are reported, including lack of knowledge about the vaccine; concerns about vaccine safety; incorrect beliefs about vaccines; belief that their children are too young for HPV vaccination; lack of health care professionals recommendation [6,9]. 

For increasing acceptability and understanding among parents, health care professionals should implement communication on HPV vaccination to increase parental awareness [5,9].

Among health care professionals, dentists could play a key role in the prevention of HPV-related OP-cancer, by educating patients and their parents on the risks associated with the HPV infection, by informing them on the HPV vaccination availability, being able to likely prevent the onset of HPV-related malignancies [13,14,15]. In addition, in October 2018, the American Dental Association encouraged dentists to support the use and the administration of the HPV vaccine, recognizing it as a way to help prevent infection of the types of HPV associated with OP-cancer [16].

Dentists are aware of their role in primary and secondary prevention of HPV- related OP-cancer and agree that they have a role in discussing HPV vaccine promotion, of which they recognize the importance [14,16]. However, as emerged in the study of Daley et al., most of them are not ready to do so due to various barriers [17]. The dentists could lack essential information on HPV disease, or they could feel uncomfortable in discussing a sexually transmitted infection with patients and their parents, or they could perceive that HPV vaccination is not within the scope of oral health professions [16]. In particular, previous studies have shown various levels of knowledge about HPV vaccination and vaccine administration among dental providers [18,19]. Some studies reported good levels of knowledge about HPV vaccine among dentists; on the other side, deficiencies regarding this topic emerged in other studies [14,15,20]. In a survey conducted by Berenson et al., nearly half of dentists mistakenly believed that HPV infection should be ruled out prior to vaccine administration and less than a third knew the number of doses to be given to patients <15 years old. In the same study, most of them recognized the need to incentivize vaccination in sexually active patients [16]. In addition, among medical providers, knowledge about HPV vaccination is poor. In a survey among Italian pediatricians, only 16.7% correctly answered how many HPV vaccines have already been, or are soon to be, registered in Europe, and only half considered 11–12-year-olds as patients eligible for vaccination [21]. However, the majority of Italian pediatricians intended to recommend HPV vaccination in both sexes, different from the findings of Daley et al., where pediatricians were in favor of vaccinating female patients rather than both genders [21,22]. A total of 33.8% of participants in a survey among pediatrics, obstetrics, and gynecology specialists in Turkey believed that the HPV vaccination should be administered only in women, and only half of pediatricians recommended vaccination to their patients [23]. As, even among medical doctors, there are no established positions on HPV vaccination, the dentist’s role in HPV vaccination counselling needs to be enhanced.

In the first systematic review on dentists’ knowledge, perception, and attitude (KAP) toward HPV vaccination and HPV-related diseases education, a hesitancy to discuss HPV and HPV vaccination with patients and a need for more education about HPV and HPV-related OP-cancer emerged [24]. Therefore, it could be difficult to achieve a role in HPV infection for dentists, because it requires public recognition and professional acceptance [25].

Furthermore, because HPV vaccination is recommended for pre-teens and young adolescents, pediatric dentists, in particular, have a unique opportunity to contribute to primary prevention of HPV-related OP-cancer by discussing the HPV vaccine with patients and their parents.

In actuality, to the best of our knowledge, in literature there are no studies that have aimed to understand whether pediatric dentists are ready to assume this responsibility, and it is not yet known what their role is in the primary prevention of the oral HPV-related diseases.

Therefore, based on these considerations, the aim of the study was to assess KAP on HPV+ OP-cancer, HPV infection, and HPV vaccination among Italian pediatric dentists.

## 2. Materials and Methods

This cross-sectional study was conducted by the Oral Medicine Unit, Department of Neuroscience, Reproductive, and Oral Sciences of the “Federico II” University of Naples (Italy), in collaboration with the “Italian Society of Paediatric Dentistry” (SIOI) and the Unesco Chair in Health Education and Sustainable Development, Paediatric Dentistry Section, “Federico II” University of Naples (Italy) between April 2020 and February 2021. The study is compliant with the ethical principles of the World Medical Association Declaration of Helsinki and it was approved by the Ethics Committee of the “Federico II” University of Naples, Italy (No. 437/20).

Paediatric dentists, members of the “Italian Society of Paediatric Dentistry” (SIOI), received, by e-mail, a link to participate in the online questionnaire via Google Forms. The consent to participate was obtained online from all the participants. In addition, participation was voluntary and the answers to the questionnaire were processed anonymously. The Google Forms survey was set with mandatory responses.

No validated instrument specifically designed to assess KAP on HPV-related OP-cancer, HPV infection, and HPV vaccination among pediatric dentists was present in the literature; therefore, the questionnaire was developed by adapting items from published surveys on the matter and by elaborating items specifically designed for this survey [15,16,21,23,25,26,27,28,29].

The questionnaire comprised four parts. The first part included questions on gender, age range, graduation year, type of practice (pediatric dentistry exclusively; pediatric dentistry not exclusively), and practice setting (academic setting; public health setting; private setting).

**Figure 1:**
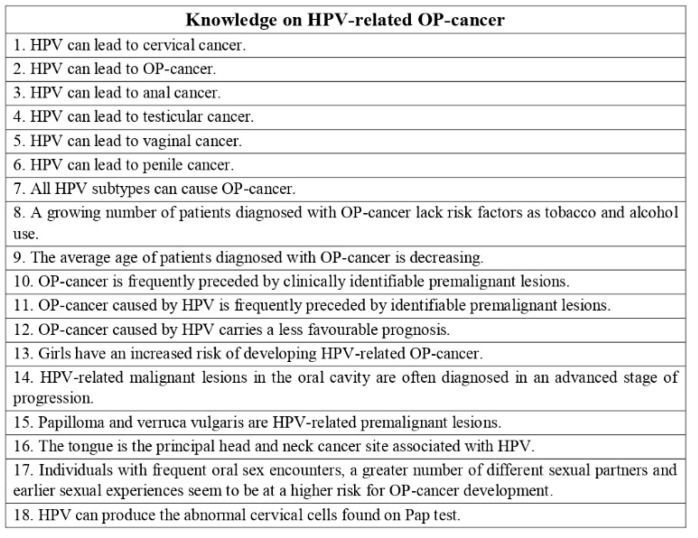
Questionnaire assessing pediatric dentists’ knowledge on HPV-related OP-cancer. Statements used required one of the following responses: “true”/“false”.

**Figure 2:**
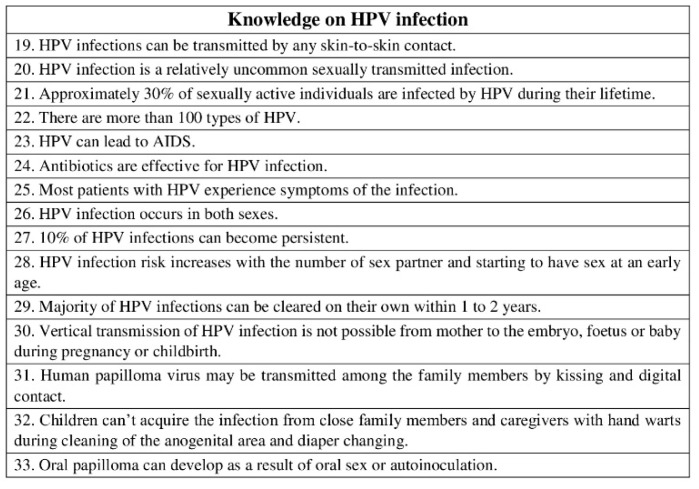
Questionnaire assessing pediatric dentists’ knowledge on HPV infection. Statements used required one of the following responses: “true”/“false”.

**Figure 3:**
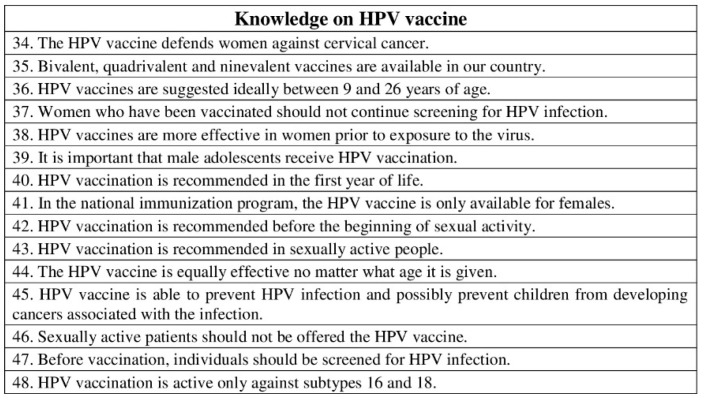
Questionnaire assessing pediatric dentists’ knowledge on HPV vaccine. Statements used required one of the following responses: “true”/“false”.

The second part consisted of 48 statements and aimed to investigate the pediatric dentists’ knowledge on HPV-related OP-cancer (statements 1–18), HPV infection (statements 19–33), and HPV vaccine (statements 34–48). Statements used required one of the following responses: “true”/“false” (Figure 1, Figure 2 and Figure 3). Then, knowledge statements were scored as correct or incorrect, with the percentage of respondents answering correctly reported in the final analysis.

**Figure 4:**
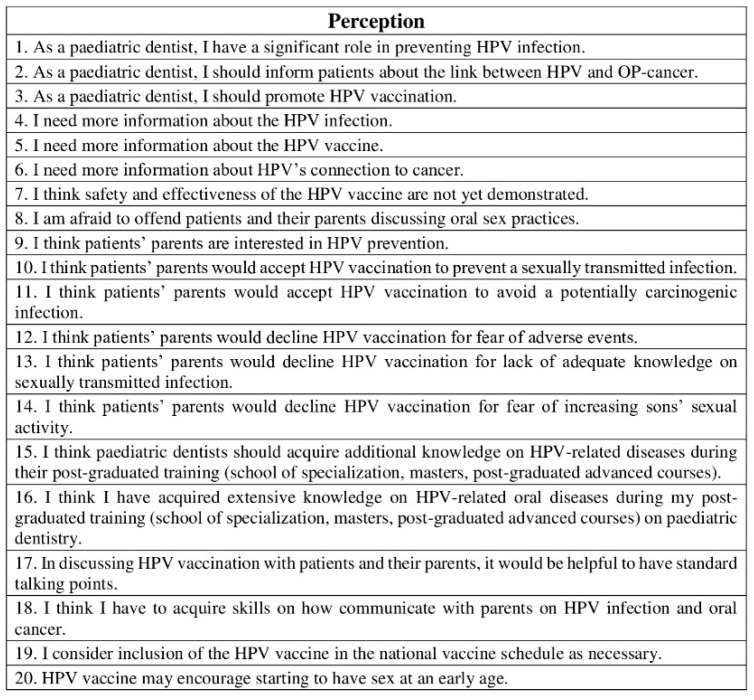
Questionnaire assessing pediatric dentists’ perceptions on HPV-related OP-cancer, HPV infection, and HPV vaccine. Level of agreement to each statement was assessed by a five-point Likert scale: 1. strongly disagree; 2. disagree; 3. neither agree nor disagree; 4. agree; 5. strongly agree.

The third part consisted of 20 statements, and it was related to the pediatric dentists’ perceptions on HPV-related OP-cancer, HPV infection, and HPV vaccine (Figure 4).

**Figure 5:**
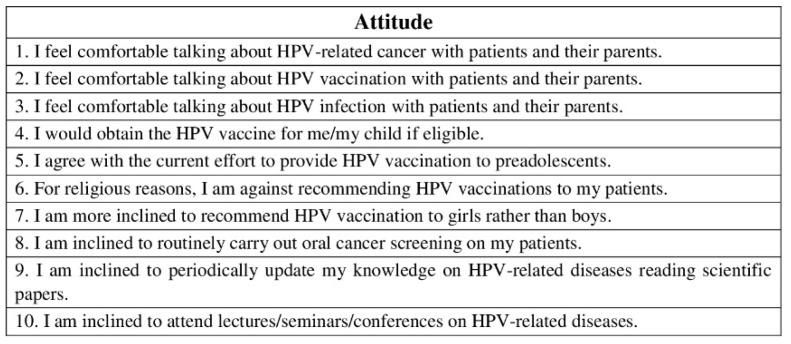
Questionnaire assessing pediatric dentists’ attitude on HPV-related OP-cancer, HPV infection, and HPV vaccine. Level of agreement to each statement was assessed by a five-point Likert scale: 1. strongly disagree; 2. disagree; 3. neither agree nor disagree; 4. agree; 5. strongly agree.

The fourth part consisted of 10 statements, and it was related to the pediatric dentists’ attitude on HPV-related OP-cancer, HPV infection, and HPV vaccine (Figure 5).

For both the third and the fourth parts, level of agreement to each statement was assessed by a five-point Likert scale: 1. strongly disagree; 2. disagree; 3. neither agree nor disagree; 4. agree; 5. strongly agree.

To assess readability, question wording, question order, and time needed to complete the questionnaire, a pilot test was performed on 10 randomly selected pediatric dentists. Minor changes were made in accordance with the results of the pilot test and incorporated into the final version of the questionnaire. The results of the pilot test were not included in the final analysis.

In addition, the questionnaire was reviewed for content validity by four experts in HPV-related OP-cancer, HPV infection, HPV vaccination, pediatric dentistry, and survey research. The experts examined the questionnaire and unanimously declared agreement with its content.

The final version of the questionnaire took approximatively 15 min to complete.

Before delivering the questionnaires, the sample size calculation for the study was performed. With a 5% margin of error and a 90% confidence level, it was calculated that, being the SIOI mailing list of around 2500, the required sample was 245.

For demographic data, both dichotomous (gender and type of practice) and non-dichotomous categorical (age range, graduation year, and practice setting), frequency distributions and relative frequencies (percentages) were calculated.

For knowledge, a comparison between ratios of correct answers for each question on the basis of different demographic characteristics was performed with Pearson’s chi-square test. For non-dichotomous categorical variables (age range, graduation year, and practice setting) a further pairwise comparison with chi-tests was performed when post hoc analysis was needed.

For attitude and practice, a comparison between frequency distribution of scores given to each question was performed with Kruskal–Wallis and Mann–Whitney test for non-dichotomous variables, and Pearson’s chi-square test for dichotomous variables.

A further comparison was performed cumulating questions on the basis of the subjects of the statements (1–18, 19–33, and 34–48, respectively).

Internal consistency for the questionnaire was estimated with Cronbach’s alpha, while to evaluate the test–retest reliability, the questionnaire was submitted twice to a group of 20 pediatric dentists, three weeks apart, using intraclass correlation coefficient (ICC) with 95% C.I. based on a mean-rating, absolute-agreement, two-way mixed-effects model.

For all statistical tests, a *p*-value less-than 0.05 was considered statistically significant. In case of multiple comparisons, *p*-values were adjusted with Bonferroni’s correction.

## 3. Results

A total of 271 pediatric dentists completed the questionnaire. The ICC ranged between good and excellent for all the statements in the questionnaire (data not reported).

The sociodemographics analysis of respondents who participated in the survey is reported in Table 1.

### 3.1. Knowledge on HPV-Related OP-Cancer (Statements 1–18)

In relation to knowledge on HPV-related OP-cancer, out of 18 questions, only 4 were incorrectly answered by more than 50% of respondents. These responders were unaware that HPV-related OP-cancer was not frequently preceded by identifiable premalignant lesions; girls did not present an increased risk of developing HPV-related OP-cancer; papilloma and verruca vulgaris were not HPV-related premalignant lesions; the tongue was not the principal head and neck cancer site associated with HPV.

Table 2 shows the percentage of correct responses on knowledge on HPV-related OP-cancer according to gender, age range, graduation year, type of practice, and practice setting.

Using chi-square test, no statistically significant differences were observed in relation to gender (*p* = 0.362), age range (*p* = 0.477), graduation year (*p* = 0.380), type of practice (*p* = 0.071), or practice setting (*p* = 0.599), respectively.

Cronbach’s alpha for this domain was 0.88, showing a good internal consistency.

### 3.2. Knowledge on HPV Infection (Statements 19–33)

In relation to knowledge on HPV infection, out of 15 questions, only 4 were incorrectly answered by more than 60% of responders. These responders did not know that HPV infections can be transmitted by any skin-to-skin contact; majority of HPV infections can be cleared on their own within 1 to 2 years; human papilloma virus may be transmitted among the family members by kissing and digital contact; the percentage of sexually active individuals infected by HPV during their lifetime was not 30%.

Table 2 shows the percentage of correct responses on knowledge on HPV infection according to gender, age range, graduation year, type of practice, and practice setting.

Chi-square test revealed that the differences were not statistically significant for gender (*p* = 0.937), age range (*p* = 0.198), graduation year (*p* = 0.393), or type of practice (*p* = 0.055), respectively; instead, they were statistically significant for practice setting (*p* = 0.001).

In particular, for practice setting, statistically significant differences were observed between public health setting and private setting (*p* = 0.004) and between academic setting and private setting (*p* = 0.006), respectively.

Cronbach’s alpha for this domain was 0.81, showing a good internal consistency.

### 3.3. Knowledge on HPV Vaccine (Statements 34–48)

In relation to knowledge on HPV vaccine, out of 15 questions, only 2 were incorrectly answered by more than 60% of responders. These responders wrongly believed that HPV vaccination is recommended in sexually active people, and before vaccination, individuals should be screened for HPV infection.

Table 2 shows the percentage of correct responses on knowledge on HPV vaccine according to gender, age range, graduation year, type of practice, and practice setting.

Using chi-square test, no statistically significant differences were observed concerning type of practice (*p* = 0.704) and practice setting (*p* = 0.280), respectively; instead, statistically significant differences were observed in relation to gender (*p* = 0.016), age range (*p* < 0.001), and graduation year (*p* = 0.002), respectively.

Specifically, regarding age range, pairwise comparison showed that the differences were statistically significant between 25–35 years and 35–45 years (*p* = 0.013), between 25–35 years and 45–55 years (*p* < 0.001), and between 25–35 years and 55–65 years (*p* = 0.017), respectively.

Relating to graduation year, pairwise chi-square test revealed that the differences were statistically significant between 1990s and 2010s (*p* < 0.001) and between 2000s and 2010s (*p* = 0.025), respectively.

Cronbach’s alpha for this domain was 0.82, showing a good internal consistency.

### 3.4. Perception

More than 80% of responders agreed or strongly agreed with the sentences stating the role of pediatric dentists in preventing HPV diseases and promoting HPV vaccination. More than 90% expressed the need to receive more information about HPV-related diseases. Only about 30% of responders stated that they were afraid to offend patients and their parents discussing oral sex practices.

Totals of 75.6% and 88.2% of the responders thought patients’ parents would accept HPV vaccination to prevent a sexually transmitted infection and to avoid a potentially carcinogenic infection, respectively. Among parental barriers to HPV vaccine acceptability, 75.3% of responders agreed or strongly agreed that patients’ parents would decline HPV vaccination for lack of adequate knowledge on sexually transmitted infection.

Almost 90% of responders agreed or strongly agreed with the sentence stating that pediatric dentists should acquire additional knowledge on HPV-related diseases during their postgraduate training.

No statistically significant differences were recorded regarding gender (*p* = 0.089); but statistically significant differences were observed in relation to age range (*p* < 0.001), graduation year (*p* = 0.007), type of practice (*p* = 0.047), and practice setting (*p* < 0.001), respectively.

Particularly, concerning age range, Mann–Whitney test indicated that the differences were statistically significant between 25–35 years and 55–65 years (*p* < 0.001), between 35–45 years and 55–65 years (*p* < 0.001), and between 45–55 years and 55–65 years (*p* < 0.001), respectively, with the 55–65 years group showing higher Likert points.

Regarding graduation year, Mann–Whitney test revealed that the differences were statistically significant between 1980s and 2000s (*p* = 0.006) and between 1980s and 2010s (*p* = 0.002), respectively, with the 1980s group showing higher Likert points.

Regarding practice setting, Mann–Whitney test demonstrated that the differences were statistically significant between academic setting and private setting (*p* = 0.013), with the academic setting group showing higher Likert points.

Cronbach’s alpha for this perception was 0.75, showing an acceptable internal consistency.

### 3.5. Attitude

More than 90% of the participants agreed or strongly agreed with the current effort to provide HPV vaccination to preadolescents; more than 80% periodically updated knowledge on HPV-related diseases, by reading scientific papers and attending lectures/seminars/conferences; almost 90% would obtain the HPV vaccine for himself/herself and his/her child.

No statistically significant differences were recorded in relation to age range (*p* = 0.174) and graduation year (*p* = 0.514), respectively; instead, statistically significant differences were observed regarding gender (*p* < 0.001), type of practice (*p* = 0.016), and practice setting (*p* = 0.007), respectively.

Specifically, in relation to practice setting, Mann–Whitney test showed that the differences were statistically significant between academic setting and private setting (*p* = 0.007), with the academic setting group showing higher Likert points.

Cronbach’s alpha for attitude was 0.72, showing an acceptable internal consistency.

Overall survey responses assessing pediatric dentists’ KAP on HPV+ OP-cancer, HPV infection, and HPV vaccination were reported in Appendix A (Table A1, Table A2, Table A3, Table A4 and Table A5).

## 4. Discussion

The present online survey aimed at assessing KAP on HPV-related OP-cancer, HPV infection, and HPV vaccination among Italian pediatric dentists. Even if the HPV vaccine is not yet approved for the prevention of OP-cancer, given the increasing incidence of this kind of malignancy, the vaccine can likely contribute to its prevention [30].

In this context, dentists could be on the front line, informing patients about the link between HPV and OP-cancer and discussing the importance of the HPV vaccine. This is even more true for pediatric dentists, considering that approximatively 85% of children aged 2–17 submit to dental visit each year [18,29].

Previous studies examined dentists’ knowledge on HPV-related diseases and HPV vaccine and investigated the possible role of dentists on HPV infection prevention through vaccine promotion in USA, reporting variable results [14,15,16,24,29,30,31]. However, to the best of our knowledge, at the time of writing, none of these studies has been focused on pediatric dentistry.

The results of the present study showed a good overall knowledge among pediatric dentists regarding HPV-related OP-cancer, HPV infection, and HPV vaccine.

In relation to knowledge on HPV infection, the percentage of correct answers about knowledge on HPV infection was statistically significant lower for private setting, when compared to both academic setting and public health setting. These results could be ascribed to dissimilar levels of awareness of the importance of sensitive topics, such as sexually transmitted infections, between different professional profiles. Notably, dentists working in private practices/clinics seemed concerned about bringing sensitive topics to patients, whereas dentists practicing in public health clinics/university hospitals described having an easier time discussing sensitive topics with patients [18].

In relation to knowledge on HPV vaccine, the percentage of correct answers about knowledge on HPV vaccine was statistically significantly higher for females when compared to males. These results could be explained considering that, even though the HPV vaccination for males has been recommended from 2017 in Italy, the issue of HPV prevention was, and currently is, largely framed as a women’s health issue [11]. Moreover, a slightly lower level of knowledge was shown by younger subjects, probably due to the lack of expertise in the field.

In summary, our results on knowledge were in line with the study of Patel et al., assessing Arizona dentists’ and dental hygienists’ knowledge and attitudes about HPV-related OP-cancer, reporting that most responders answered HPV knowledge questions correctly [15]. In contrast, a very recent study assessing knowledge of Chinese dentists on HPV showed scant knowledge on the matter [32].

In addition to knowledge, our survey also investigated perception of the role of pediatric dentists on prevention of HPV-related diseases. Our results showed that the large portion of the participants believed that they had a significant role in preventing HPV infection, informing patients about the link between HPV and OP-cancer and promoting HPV vaccination. This was in line with a German study by Poleman et al., reporting that dental professionals felt they had a clear role and responsibility in discussing the relation between oral cancer and HPV with patients [25].

However, as reported in other studies conducted in USA and in Malaysia [24,33], most of the responders felt the need for more information about HPV’s connection to cancer, HPV infection, and HPV vaccine; they thought pediatric dentists should acquire additional knowledge on HPV-related diseases during their postgraduate training, and should acquire skills on how to communicate with parents on HPV infection and oral cancer; they would have standard talking points in discussing HPV vaccination with patients and their parents. One reason for these requests may be that most pediatric dentists do not usually receive HPV-related education in their postgraduate formation. In fact, in a study carried out in USA, assessing what is currently being taught in postgraduate pediatric dental programs, regarding HPV infection, HPV vaccine, and risk factors associated with OP-cancer, it emerged that only 25% of program directors of postgraduate pediatric dental programs included information about HPV and HPV vaccine in their curricula [28]. This need can be addressed through improved educational training programs to provide pediatric dentists with the informational tools and education required to discuss HPV with patients and their parents [14,26].

Therefore, lack of knowledge on the subject was the main barrier for dentists to discuss HPV with their patients; however, findings from previous study, conducted in USA, showed that another barrier to discuss HPV was represented by the lack of privacy and the fear of offending patients [14]. Another concern for dentists was patient demographics. Most dentists felt comfortable discussing HPV and vaccination with their 18-year-old or older patients, but only a minority felt comfortable with even younger patients (9–12 years old) [16]. In addition to age, gender also represented a barrier to discussing vaccination. In particular, male dentists described discomfort in discussing HPV with female patients, and the majority of dental practitioners had no intention of doing so [17,31].

In a survey conducted among members of American Dental Association Clinical Evaluators, only 1/4 discussed HPV vaccination with patients or parents. In fact, they believed that it was more appropriate to delegate discussions on this topic to other health professionals; they did not know how to approach the topic and they were not comfortable discussing sexually transmitted infections. Furthermore, 38% of respondents found it difficult to administer the vaccine or did not feel comfortable doing so, both for obtaining reimbursement and for vaccine preservation [34].

On pediatric dentists’ attitude on HPV-related OP-cancer, HPV infection, and HPV vaccination, our results were quite interesting. In fact, as mentioned before, while in the literature most of the studies from USA reported that dentists described discomfort in having sexual health-related discussions with patients and parents [17,30], most of the pediatric dentists participating in the present survey demonstrated a positive or neutral attitude in talking about HPV-related cancer, HPV vaccination, and HPV infection with patients and their parents.

The present study had certain limitations that should be addressed. First of all, the participation to this survey being voluntary, subjects who decided to participate may have been conditioned by former knowledge on the topic of HPV. Second, when using this kind of survey, there is the risk that responders are likely to answer in the way they consider would be more appropriate for the survey writers [26]. Third, among responders, females were overrepresented. However, as highlighted by Daley et al., the overrepresentation of women in voluntary survey is common in most fields and cannot be interpreted as having any inferential meaning [31].

On the other hand, online surveys have an advantage, when compared to paper-based surveys. In fact, in paper-based surveys, responders can skip questions, leading to incomplete results; instead, online surveys require participants to complete answers before continuing to the next question. In this way, the risk of incomplete data can be cleared [14].

## 5. Conclusions

To conclude, this is the first study aiming to assess KAP on HPV-related OP-cancer, HPV infection, and HPV vaccination among pediatric dentists. Findings from the current study are encouraging, because pediatric dentists participating in the survey showed an overall satisfying knowledge on HPV-related diseases and HPV prevention through vaccine promotion. Furthermore, they manifested a good attitude in discussing sensitive topics with patients and their parents. However, responders expressed the need for acquiring more information on the matter and skills on how to communicate with patients and their parents on HPV infection and oral cancer. Improving educational training programs, as well as informing about prevention of HPV-related OP-cancer, exploring continuing professional development modules, and designing and sharing of resources/tools specifically for the use of already-practicing pediatric dentists, will place them on the front line to counteract HPV-related diseases.

## Figures and Tables

**Table 1:** Sociodemographics analysis of respondents who participated in the survey N = 271.

Characteristics	N (%)
**Gender** Male	64 (23.6)
Female	207 (76.4)
**Age range** 25–35 years	116 (42.8)
35–45 years	85 (31.4)
45–55 years	44 (16.2)
55–65 years	26 (9.6)
**Graduation year** 1970s	2 (0.7)
1980s	20 (7.4)
1990s	41 (15.1)
2000s	86 (31.7)
2010s	122 (45.0)
**Type of practice** Paediatric dentistry exclusively	37 (13.7)
Paediatric dentistry not exclusively	234 (86.3)
**Practice setting** Academic setting	33 (12.2)
Public health setting	10 (3.7)
Private setting	228 (84.1)

**Table 2:** Percentages of correct responses on knowledge on HPV-related OP-cancer, HPV infection, and HPV vaccine, according to gender, age range, graduation year, type of practice, and practice setting.

Characteristics	Knowledge on HPV-Related OP-Cancer (Correct Responses)	Knowledge on HPV Infection (Correct Responses)	Knowledge on HPV Vaccine (Correct Responses)
**Gender**			
Male	68%	68.4%	74.0%
Female	69.5%	68.5%	77.9%
**Age range**			
25–35 years	69.9%	69.1%	73.9%
35–45 years	68.8%	67.3%	77.9%
45–55 years	69.5%	70.9%	81.5%
55–65 years	68.4%	65.3%	79.7%
**Graduation year**			
1970s	72.2%	63.3%	70.0%
1980s	66.6%	66.3%	79.6%
1990s	69.2%	70.5%	81.6%
2000s	67.2%	67.0%	77.9%
2010s	70.4%	69.3%	74.4%
**Type of practice**			
Paediatric dentistry exclusively	72.2%	72.1%	77.6%
Paediatric dentistry not exclusively	68.7%	68.0%	76.8%
**Practice setting**			
Academic setting	70.9%	73.5%	75.1%
Public health setting	70.0%	78.6%	81.3%
Private setting	68.9%	56.6%	77.0%

## Data Availability

Data is contained within the article.

## Appendix A

**Table A1:** Survey responses assessing pediatric dentists’ knowledge on HPV-related OP-cancer. Statements used required one of the following responses: “true”/“false”. The correct answer is bold and underlined.

Knowledge on HPV-Related OP-Cancer			
Statements		Correct N (%)	Incorrect N (%)
**1.**	HPV can lead to cervical cancer. **True**/False	269 (99.3)	2 (0.7)
**2.**	HPV can lead to OP-cancer. **True**/False	268 (98.9)	3 (1.1)
**3.**	HPV can lead to anal cancer. **True**/False	158 (58.3)	113 (41.7)
**4.**	HPV can lead to testicular cancer. True/**False**	204 (75.3)	67 (24.7)
**5.**	HPV can lead to vaginal cancer. **True**/False	158 (58.3)	113 (41.7)
**6.**	HPV can lead to penile cancer. **True**/False	137 (50.6)	134 (49.4)
**7.**	All HPV subtypes can cause OP-cancer. True/**False**	197 (72.7)	74 (27.3)
**8.**	A growing number of patients diagnosed with OP-cancer lack risk factors as tobacco and alcohol use. **True**/False	239 (88.2)	32 (11.8)
**9.**	The average age of patients diagnosed with OP-cancer is decreasing. **True**/False	217 (80.1)	54 (19.9)
**10.**	OP-cancer is frequently preceded by clinically identifiable premalignant lesions. **True**/False	237 (87.5)	34 (12.5)
**11.**	OP-cancer caused by HPV is frequently preceded by identifiable premalignant lesions. True/**False**	60 (22.1)	211 (77.9)
**12.**	OP-cancer caused by HPV carries a less favorable prognosis. True/**False**	155 (57.2)	116 (42.8)
**13.**	Girls have an increased risk of developing HPV-related OP-cancer. True/**False**	135 (49.8)	136 (50.2)
**14.**	HPV-related malignant lesions in the oral cavity are often diagnosed in an advanced stage of progression. **True**/False	220 (81.2)	51 (18.8)
**15.**	Papilloma and verruca vulgaris are HPV-related premalignant lesions. True/**False**	66 (24.4)	205 (75.6)
**16.**	The tongue is the principal head and neck cancer site associated with HPV. True/**False**	131 (48.3)	140 (51.7)
**17.**	Individuals with frequent oral sex encounters, a greater number of different sexual partners and earlier sexual experiences seem to be at a higher risk for OP-cancer development. **True**/False	259 (95.6)	12 (4.4)
**18.**	HPV can produce the abnormal cervical cells found on Pap test. **True**/False.	264 (97.4)	7 (2.6)

**Table A2:** Survey responses assessing pediatric dentists’ knowledge on HPV infection. Statements used required one of the following responses: “true”/“false”. The correct answer is bold and underlined.

Knowledge on HPV Infection			
Statements		Correct N (%)	Incorrect N (%)
**19.**	HPV infections can be transmitted by any skin-to-skin contact. **True**/False	67 (24.7)	204 (75.3)
**20.**	HPV infection is a relatively uncommon sexually transmitted infection. True/**False**	173 (63.8)	98 (36.2)
**21.**	Approximately 30% of sexually active individuals are infected by HPV during their lifetime. True/**False**	46 (17.0)	225 (83.0)
**22.**	There are more than 100 types of HPV. **True**/False	217 (80.1)	54 (19.9)
**23.**	HPV can lead to AIDS. True/**False**	261 (96.3)	10 (3.7)
**24.**	Antibiotics are effective for HPV infection. True/**False**	262 (96.7)	9 (3.3)
**25.**	Most patients with HPV experience symptoms of the infection. True/**False**	235 (86.7)	36 (13.3)
**26.**	HPV infection occurs in both sexes. **True**/False	253 (93.4)	18 (6.6)
**27.**	10% of HPV infections can become persistent. **True**/False	251 (92.6)	20 (7.4)
**28.**	HPV infection risk increases with the number of sex partner and starting to have sex at an early age. **True**/False	258 (95.2)	13 (4.8)
**29.**	Majority of HPV infections can be cleared on their own within 1 to 2 years. **True**/False	102 (37.6)	169 (62.4)
**30.**	Vertical transmission of HPV infection is not possible from mother to the embryo, fetus or baby during pregnancy or childbirth. True/**False**	179 (66.1)	92 (33.9)
**31.**	Human papilloma virus may be transmitted among the family members by kissing and digital contact. **True**/False	77 (28.4)	194 (71.6)
**32.**	Children cannot acquire the infection from close family members and caregivers with hand warts during cleaning of the anogenital area and diaper changing. True/**False**	160 (59.0)	111 (41.0)
**33.**	Oral papilloma can develop as a result of oral sex or autoinoculation. **True**/False	245 (90.4)	26 (9.6)

**Table A3:** Survey responses assessing pediatric dentists’ knowledge on HPV vaccine. Statements used required one of the following responses: “true”/“false”. The correct answer is bold and underlined.

Knowledge on HPV Vaccine			
Statements		Correct N (%)	Incorrect N (%)
**34.**	The HPV vaccine defends women against cervical cancer. **True**/False	258 (95.2)	13 (4.8)
**35.**	Bivalent, quadrivalent, and nonavalent vaccines are available in our country. **True**/False	202 (74.5)	69 (25.5)
**36.**	HPV vaccines are suggested ideally between 9 and 26 years of age. **True**/False	258 (95.2)	13 (4.8)
**37.**	Women who have been vaccinated should not continue screening for HPV infection. True/**False**	254 (93.7)	17 (6.3)
**38.**	HPV vaccines are more effective in women prior to exposure to the virus. **True**/False	248 (91.5)	23 (8.5)
**39.**	It is important that male adolescents receive HPV vaccination. **True**/False	210 (77.5)	61 (22.5)
**40.**	HPV vaccination is recommended in the first year of life. True/**False**	257 (94.8)	14 (5.2)
**41.**	In the national immunization program, the HPV vaccine is only available for females. True/**False**	147 (54.2)	124 (45.8)
**42.**	HPV vaccination is recommended before the beginning of sexual activity. **True**/False	259 (95.6)	12 (4.4)
**43.**	HPV vaccination is recommended in sexually active people. True/**False**	100 (36.9)	171 (63.1)
**44.**	The HPV vaccine is equally effective no matter what age it is given. True/**False**	179 (66.1)	92 (33.9)
**45.**	HPV vaccine is able to prevent HPV infection and possibly prevent children from developing cancers associated with the infection. **True**/False	241 (88.9)	30 (11.1)
**46.**	Sexually active patients should not be offered the HPV vaccine. True/**False**	246 (90.8)	25 (9.2)
**47.**	Before vaccination, individuals should be screened for HPV infection. True/**False**	107 (39.5)	164 (60.5)
**48.**	HPV vaccination is active only against subtypes 16 and 18. True/**False**	163 (60.1)	108 (39.9)

**Table A4:** Survey responses assessing pediatric dentists’ perception on HPV-related OP-cancer, HPV infection and HPV vaccine. Each of these statements can be answered on a five-point scale: 1. strongly disagree; 2. disagree; 3. neither agree nor disagree; 4. agree; 5. strongly agree.

Perception						
Statements		Strongly Disagree N (%)	Disagree N (%)	Neither Agree Nor Disagree N (%)	Agree N (%)	Strongly Agree N (%)
**1.**	As a pediatric dentist, I have a significant role in preventing HPV infection.	1 (0.4)	2 (0.7)	38 (14.0)	127 (46.9)	103 (38.0)
**2.**	As a pediatric dentist, I should inform patients about the link between HPV and OP-cancer.	3 (1.1)	5 (1.8)	30 (11.1)	127 (46.9)	106 (39.1)
**3.**	As a pediatric dentist, I should promote HPV vaccination.	3 (1.1)	4 (1.5)	41 (15.1)	117 (43.2)	106 (39.1)
**4.**	I need more information about the HPV infection.	4 (1.5)	4 (1.5)	18 (6.6)	123 (45.4)	122 (45.0)
**5.**	I need more information about the HPV vaccine.	2 (0.7)	2 (0.7)	14 (5.2)	136 (50.2)	117 (43.2)
**6.**	I need more information about HPV’s connection to cancer.	2 (0.7)	15 (5.5)	27 (10.0)	137 (50.6)	90 (33.2)
**7.**	I think safety and effectiveness of the HPV vaccine are not yet demonstrated.	73 (26.9)	116 (42.8)	58 (21.4)	18 (6.6)	6 (2.2)
**8.**	I am afraid to offend patients and their parents discussing oral sex practices.	24 (8.9)	73 (26.9)	89 (32.8)	71 (26.2)	14 (5.2)
**9.**	I think patients’ parents are interested in HPV prevention.	1 (0.4)	13 (4.8)	45 (16.6)	166 (61.3)	46 (17.0)
**10.**	I think patients’ parents would accept HPV vaccination to prevent a sexually transmitted infection.	4 (1.5)	10 (3.7)	52 (19.2)	164 (60.5)	41 (15.1)
**11.**	I think patients’ parents would accept HPV vaccination to avoid a potentially carcinogenic infection.	0	3 (1.1)	29 (10.7)	162 (59.8)	77 (28.4)
**12.**	I think patients’ parents would decline HPV vaccination for fear of adverse events.	7 (2.6)	80 (29.5)	103 (38.0)	70 (25.8)	11 (4.1)
**13.**	I think patients’ parents would decline HPV vaccination for lack of adequate knowledge on sexually transmitted infection.	5 (1.8)	18 (6.6)	44 (16.2)	140 (51.7)	64 (23.6)
**14.**	I think patients’ parents would decline HPV vaccination for fear of increasing sons’ sexual activity.	22 (8.1)	98 (36.2)	95 (35.1)	43 (15.9)	13 (4.8)
**15.**	I think pediatric dentists should acquire additional knowledge on HPV-related diseases during their post-graduated training (school of specialization, masters, post-graduated advanced courses).	3 (1.1)	6 (2.2)	21 (7.7)	133 (49.1)	108 (39.9)
**16.**	I think I have acquired extensive knowledge on HPV-related oral diseases during my post-graduated training (school of specialization, masters, post-graduated advanced courses) on pediatric dentistry.	20 (7.4)	121 (44.6)	72 (26.6)	52 (19.2)	6 (2.2)
**17.**	In discussing HPV vaccination with patients and their parents, it would be helpful to have standard talking points.	2 (0.7)	3 (1.1)	45 (16.6)	175 (64.6)	46 (17.0)
**18.**	I think I have to acquire skills on how communicate with parents on HPV infection and oral cancer.	4 (1.5)	21 (7.7)	40 (14.8)	154 (56.8)	52 (19.2)
**19.**	I consider inclusion of the HPV vaccine in the national vaccine schedule as necessary.	1 (0.4)	2 (0.7)	20 (7.4)	155 (57.2)	93 (34.3)
**20.**	HPV vaccine may encourage starting to have sex at an early age.	85 (31.4)	129 (47.6)	42 (15.5)	12 (4.4)	3 (1.1)

**Table A5:** Survey responses assessing pediatric dentists’ attitude on HPV-related OP-cancer, HPV infection, and HPV vaccine. Each of these statements can be answered on a five-points scale: 1. strongly disagree; 2. disagree; 3. neither agree nor disagree; 4. agree; 5. strongly agree.

Attitude						
Statements		Strongly Disagree N (%)	Disagree N (%)	Neither Agree Nor Disagree N (%)	Agree N (%)	Strongly Agree N (%)
**1.**	I feel comfortable talking about HPV-related cancer with patients and their parents.	8 (3.0)	39 (14.4)	92 (33.9)	107 (39.5)	25 (9.2)
**2.**	I feel comfortable talking about HPV vaccination with patients and their parents.	8 (3.0)	25 (9.2)	70 (25.8)	138 (50.9)	30 (11.1)
**3.**	I feel comfortable talking about HPV infection with patients and their parents.	7 (2.6)	28 (10.3)	77 (28.4)	130 (48.0)	29 (10.7)
**4.**	I would obtain the HPV vaccine for me/my child if eligible.	3 (1.1)	3 (1.1)	22 (8.1)	143 (52.8)	100 (36.9)
**5.**	I agree with the current effort to provide HPV vaccination to preadolescents.	1 (0.4)	1 (0.4)	21 (7.7)	139 (51.3)	109 (40.2)
**6.**	For religious reasons, I am against recommending HPV vaccinations to my patients.	180 (66.4)	74 (27.3)	8 (3.0)	9 (3.3)	0
**7.**	I am more inclined to recommend HPV vaccination to girls rather than boys.	55 (20.3)	89 (32.8)	60 (22.1)	61 (22.5)	6 (2.2)
**8.**	I am inclined to routinely carry out oral cancer screening on my patients.	4 (1.5)	13 (4.8)	48 (17.7)	134 (49.4)	72 (26.6)
**9.**	I am inclined to periodically update my knowledge on HPV-related diseases reading scientific papers.	2 (0.7)	12 (4.4)	32 (11.8)	147 (54.2)	78 (28.8)
**10.**	I am inclined to attend lectures/seminars/conferences on HPV-related diseases.	1 (0.4)	4 (1.5)	33 (12.2)	137 (50.6)	96 (35.4)
